# MEK1/2 Inhibitor (GDC0623) Promotes Osteogenic Differentiation of Primary Osteoblasts Inhibited by IL-1*β* through the MEK-Erk1/2 and Jak/Stat3 Pathways

**DOI:** 10.1155/2021/5720145

**Published:** 2021-12-22

**Authors:** Zeng-Qiao Zhang, Xiao-Shen Hu, Ye-Chen Lu, Jun-Peng Zhang, Wen-Yao Li, Wei-Yang Zhang, Wei Feng, Dao-Fang Ding, Jian-Guang Xu

**Affiliations:** ^1^School of Rehabilitation Science, Shanghai University of Traditional Chinese Medicine, Shanghai, China; ^2^School of Health Preservation and Rehabilitation, Chengdu University of Traditional Chinese Medicine, Chengdu, China; ^3^School of Sports Medicine and Health, Chengdu Sport University, Chengdu, China

## Abstract

**Objective:**

We evaluated the effects and mechanisms of GDC0623 on osteogenic differentiation of osteoblasts induced by IL-1*β*. *Methodology*. Osteoblasts were treated with 20 ng/ml IL-1*β* and 0.1 *µ*M GDC0623. Cell proliferation levels were evaluated by the cell counting kit 8 (CCK8), EdU assay, and western blotting [proliferating cell nuclear antigen (PCNA) and Cyclin D1]. Osteoblasts were cultured in an osteogenic induction medium for 1–3 weeks after which their differentiations were assessed by alkaline phosphatase (ALP) staining, Alizarin Red staining, calcium concentration, immunocytochemistry staining, real-time quantitative PCR (RT-qPCR), and immunofluorescence staining. The osteogenesis-associated mechanisms were further evaluated by western blotting using appropriate antibodies.

**Results:**

Relative to the control group, IL-1*β* induced the rapid proliferation of osteoblasts and suppressed their osteogenic differentiations by upregulating the activities of MEK-Erk1/2 as well as Jak-Stat3 pathways and by elevating MMP13 and MMP9 levels. However, blocking of the MEK-Erk1/2 signaling pathway by GDC0623 treatment reversed these effects.

**Conclusion:**

Inhibition of Jak-Stat3 pathway by C188-9 downregulated the expression levels of MMP9 and MMP13, activated MEK-Erk1/2 pathway, and inhibited osteogenic differentiation.

## 1. Introduction

Osteoporosis (OP), a common disease in postmenopausal women and the elderly, is characterized by decreased bone mass, abnormal bone structure, and increased bone fragility [1]. The increasing incidence rates of osteoporosis are a global public health problem [2]. Osteoporotic fractures are associated with increased mortality rates, decreased quality of life, and a high socioeconomic burden. Currently, studies are investigating potential preventive and cure options for osteoporosis.

Initially, osteoporosis was defined as a nutritional, metabolic, and endocrine disease. Currently, it is defined as a chronic low-grade inflammatory disease [3]. The occurrence and development of osteoporosis, especially primary osteoporosis, are directly associated with inflammation [4–6]. Proinflammatory cytokines, such as tumor necrosis factor alpha (TNF-*α*), interleukin-1*β* (IL-1*β*), and interleukin-6 (IL-6) are principal mediators of inflammation. These cytokines break the balance of bone turnover by either reducing bone formation of osteoblasts or increasing bone absorption of osteoclasts [7, 8]. IL-1*β* is a pathogenetic factor in inflammation-related degenerative diseases [9, 10]. Therefore, anti-inflammatory approaches have become potential therapeutic strategies for preventing and treating OP.

IL-1*β* plays a negative role in the pathogenesis of inflammatory-associated bone metabolism diseases, such as rheumatoid arthritis (RA) and bone fractures. IL-1*β*-mediates MAPK activation in inflammatory environments and inhibits BMP2-induced ALP activity, calcium deposition, and expressions of osteogenic markers in C2C12 and MC3T3-E1 cells [11, 12]. ERK1/2 promotes STAT3 phosphorylation [13], and crosstalk between STAT3 and MEK-Erk1/2 pathways in other tissues have been reported [14–16]. The ERK1/2 signaling pathway has been extensively studied in various diseases. However, it has not been established whether osteogenic differentiation is affected by ERK1/2 activation.

The pathogenesis of osteoporosis involves excessive bone resorption by osteoclasts, which is regulated by osteoblasts. Antiosteoporotic drugs inhibit bone resorption; however, they exert modest effects on anabolic bone formation. Therefore, there is a need to elucidate the underlying mechanisms involved in inflammation-induced inhibition of osteogenic differentiation and to develop an efficient drug with the ability to regulate osteogenic differentiation under inflammatory conditions.

In this study, osteoblast proliferation was promoted by IL-1*β* treatment, which inhibited their differentiation. The MEK inhibitor, GDC0623, significantly reversed the suppressive effects of IL-1*β* on osteoblastic differentiation, including ALP activities, formation of mineralized bones, and expression levels of osteoblast-specific genes. Protein levels of p-Stat3, MMP13, and MMP9 were downregulated by inhibition of the MEK-Erk1/2 and Jak2-Stat3 pathways, respectively. Inhibition of Stat3 with a specific inhibitor, C188-9, enhanced Erk1/2 phosphorylation levels but suppressed osteogenic differentiation.

## 2. Materials and Methods

### 2.1. Culture of Primary Osteoblasts and Intervention Methods

Primary osteoblasts were isolated from the calvaria of neonatal Sprague-Dawley (SD) rats (SIPPR-BK Inc., Shanghai, China) and digested using 0.1% collagenase type I in DMEM medium (Sigma-Aldrich, Saint Louis, MO, USA) in a shaking bath at 37°C for 60 min. Osteoblasts were cultured in Dulbecco's Modified Eagle's Medium (DMEM, Biowest, France) supplemented with 10% fetal bovine serum (FBS, Biowest, France). Osteoblasts at passage P1 were used for phenotypic and functional studies. To assess protein phosphorylation levels, osteoblasts were cultured in DMEM plus 0.1% FBS overnight and treated with 20 ng/ml IL-1*β* (RD company) or IL-1*β* plus 0.1 *μ*M GDC0623 (Selleck, China) or IL-1*β* plus 5 *μ*M C188-9 (Selleck, China) for 60 min.

### 2.2. Osteogenic Differentiation and Experimental Groups

Osteoblastic cells were seeded in 6-well plates and incubated to 75% confluence. After 24 h following passaging, the maintenance medium was replaced with osteogenic induction differentiation medium kit (Cyagen, China). The medium kit consisted of the culture medium, 10% FBS, 10 mM *β*-glycerophosphate, 50 *μ*g/ml L-ascorbic acid, and 10-7 M dexamethasone. The medium was replaced after every 3 days. Osteoblasts were divided into three groups: control group (osteoblasts were cultured in the osteogenic induction differentiation medium), model group (osteoblasts were cultured in the osteogenic induction differentiation medium + 20 ng/ml IL-1*β*), and GDC0623 group (osteoblasts were cultured in osteogenic induction differentiation medium + 20 ng/ml IL-1*β* + 0.1 *μ*M GDC0623). After culturing for one or three weeks, osteoblasts were induced for further assays.

### 2.3. EdU Assay

Cells were incubated in DMEM supplemented with 10 *μ*M EdU (Cat. no. C10310, RiboBio, China) for 6 h. Then, they were washed using phosphate-buffered saline (PBS) and fixed in 4% paraformaldehyde. Cells were incubated in the presence of glycine (2 mg/ml), washed using PBS twice, and permeabilized using PBS containing 0.5% triton X-100. After washing with PBS, cells were incubated for 30 min in the presence of Apollo® staining solution and washed thrice using PBST (PBS containing 0.5% Triton X-100), followed by 10 min of incubation in the presence of Hoechst. Cells were imaged using the Olympus IX73 fluorescent microscope (Olympus, Tokyo, Japan).

### 2.4. Assessment of ALP Activities

Osteogenically differentiated cells were obtained after 7 days of cultivation in the osteogenic medium. Protein concentrations were determined using the bicinchoninic acid (BCA) assay kit (Cat. no. 23227, Pierce, USA). A total of 20 *µ*g proteins were mixed with 100 *µ*L pNPP substrate 1-step TM PNPP (Cat. no 37621, Pierce, USA) and incubated at room temperature for 30 min. Reactions were terminated with 50 *µ*L of 2 M NaOH. The product, a yellow p-nitro phenol (pNP) compound, was measured at a wavelength of 405 nm.

### 2.5. Alizarin Red Staining

After 21 days of osteogenic differentiation, osteoblasts were fixed in 4% paraformaldehyde (PFA) at room temperature for 20 min, incubated with Alizarin Red S staining solution (Cyagen, China) for 20 min, followed by 3 extensive washes using PBS to visualize calcium formation.

### 2.6. The Calcium Colorimetric Assay Kit

Calcium ion content was determined using the calcium colorimetric assay kit (Cat. no. S1063S, Beyotime, China). The supernatant was discarded, after which cells were washed once using PBS. The lysis buffer, at a ratio of 100–200 *μ*l, was added to each well of a 6-well culture plate. Cells were fully lysed on ice. After lysis, cells were centrifuged at 14,000*g* at 4°C for 5 min. The same amount of protein was detected. Microplate reader (BioTek microplate reader) was used to measure the absorbance at 575 nm. The standard solution and curve were obtained according to kit instructions.

### 2.7. Real-Time Quantitative PCR (RT-qPCR)

Total RNA was extracted from osteoblasts using the RNA extraction kit (Cat. no. 9767, Takara, Japan) in accordance with the manufacturer's instructions. The residual DNA was removed by DNase treatment with DNase I (Cat. no. D2215, Takara, Japan). The purity of the RNA was measured with a UV-Vis spectrophotometer (NanoDrop Lite, Thermo scientific, USA) at 260 and 280 nm wavelengths. First strand cDNA of RNA samples with purity ratios (an average 260/280) between 1.8 and 2.0 was synthesized using the RT Reagent Kit (RR036Q, Takara, Japan). Quantitative real-time polymerase chain reaction (qPCR) was completed using the SYBR qPCR Master Mix (B21203, Bimake, China). The PCR was performed under the following conditions: initial denaturation at 95°C for 30 sec, 40 cycles of two-step PCR (95°C for 5 sec and 60°C for 30 sec), and dissociation (95°C for 15 sec, 60°C for 1 min, and 95°C for 15 sec). For contamination assessment, we launched PCR analysis with no-RT controls (without RT enzyme). This experiment was performed in triplicates. The ΔΔ Ct method was used to analyze target genes. GAPDH was used as a reference gene. The primers used in this study are shown in Table 1.

### 2.8. Western Blotting

Supernatants were collected for determination of total proteins after cells had been lysed on ice using the RIPA lysis buffer containing 10% phenylmethanesulfonyl fluoride. Protein concentrations were determined using the BCA Protein Assay Kit (Cat. no. 23227, Pierce, USA). Then, they were separated using 10% SDS-PAGE and transferred to a PVDF membrane. The membrane was incubated with primary antibodies and probed with respective secondary antibodies. The following antibodies were used: Cyclin D1(#2978, CST, USA), PCNA (SC-25280, USA), P-Erk1/2(#9101, CST, USA), Erk1/2(#4695, CST, USA), Stat3(#4904, CST, USA), P-Stat3(#9145, CST, USA), P-Stat3(#9134, CST, USA), MMP13(SC-30073, USA), MMP9(A5725, Bimake, China) and GAPDH (#2118s, CST, USA).

### 2.9. Immunofluorescence Assay

Osteoblasts were cultured on coverslips and maintained in a culture medium. They were fixed in 4% paraformaldehyde at room temperature for 10 min and permeabilized for 10 min using phosphate buffered saline-Tween 20 (PBS +0.1% Triton X-100). Then, cells were incubated overnight in the presence of primary antibodies against Runx2 (AF2593, Beyotime, China) at 4°C after being blocked with 5% BSA for 1h, washed thrice using PBST and stained with a fluorescent secondary antibody in the dark for 30 min. Finally, the nucleus was stained using 10 ng/ml DAPI for 5 min after which cells were observed under fluorescence microscopy (Cat. no. A1001, Applichem, Germany). Negative control experiment (only PBS instead of the primary antibody was added to the specimen) was conducted to ensure the specificity of immunofluorescence histochemical staining and exclude nonspecific staining.

### 2.10. Immunocytochemistry

Osteoblasts from each group were fixed on slides, fixed in 4% paraformaldehyde at room temperature for 10 min, and rinsed using the phosphate buffer (PBS). Slides were incubated with 0.1% Triton X-100 for 20 min and rinsed using PBS. Endogenous peroxidase was blocked with enhanced endogenous peroxidase blocking buffer (Beyotime, China) for 10 min. Slides were incubated in the blocking solution for 10 min (QuickBlock™ kit, Beyotime), followed by overnight incubation at 4°C in the presence of primary antibodies, ColI (ARG21965, Taiwan, China), and OCN (AF6297, Beyotime, China). Then, they were washed thrice using PBS and incubated with a secondary antibody in 1% BSA for 30 min at room temperature. Diaminobenzidine (DAB) was used as the substrate for color development. Nuclei were stained with hematoxylin to mark all cells and observed under an inverted microscope. Negative control experiment (only PBS instead of the primary antibody was added to the specimen) was conducted to ensure the specificity of immunocytochemistry staining and exclude nonspecific staining.

### 2.11. Cell Counting Kit (CCK-8) Assay

Cell proliferation was assessed using the CCK8 assay (Dojindo, Japan). Osteoblasts were seeded in 96-well plates (3000 cells/well) and divided into three groups as described above (control, model, and pharmacological intervention groups). The medium was replaced with the desired osteogenic differentiation medium as described above. Cells were further incubated for 24, 48, and 72 h. Absorbance at 450 nm was measured using a microplate reader (BioTek microplate reader). These experiments were performed in triplicates.

### 2.12. Statistical Analysis

Data are expressed as mean ± SD for *n* = 3. Statistical analyses were conducted using SPSS 23.0. Analysis of variance was used to compare means among groups while pairwise comparisons were performed by the SNK method. *p* ≤ 0.05 were considered statistically significant.

## 3. Results

### 3.1. Effects of IL-1*β* on the Alkaline Phosphatase (ALP) Activities in Differentiated Osteoblasts

**Figure 1:**
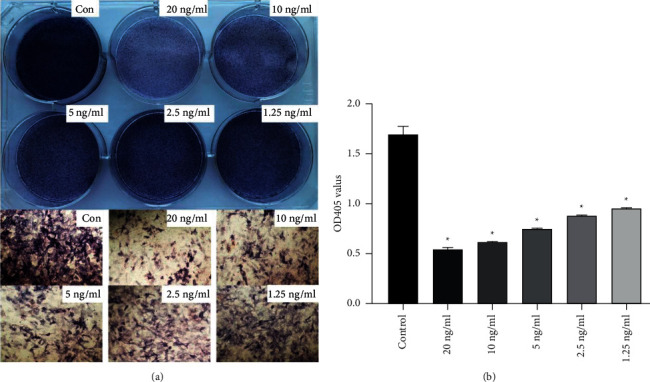
Comparisons of ALP staining and activities between the control and IL-1*β* treatment groups (20 ng/ml, 10 ng/ml, 5 ng/ml, 2.5 ng/ml, and 1.25 ng/ml). (a) Osteoblasts were stained for ALP levels after being induced with the osteogenic differentiation medium for 7 days (upper lanes). Details of ALP staining at day 7 (lower lanes). (b) ALP activities were detected after cell induction for 7 days. Data are expressed as mean ± SD for *n* = 3. ^*∗*^*p* < 0.05 versus the control group.

Alkaline phosphatase (ALP) activities were found to be upregulated in the early stages of osteogenic differentiation. After 7 days of osteogenic induction, ALP staining showed that different concentrations of IL-1*β* inhibited ALP signals, compared to the control group (Figure 1(a)). Quantitation of ALP activities revealed that each concentration of IL-1*β* inhibited cell osteogenic differentiation. The ALP activities of osteoblasts were the lowest in the 20 ng/ml IL-1*β* treatment group (Figure 1(b)).

### 3.2. Effects of GDC0623 on Alkaline Phosphatase (ALP) Activities in IL-1*β*-Induced Differentiated Osteoblasts

**Figure 2:**
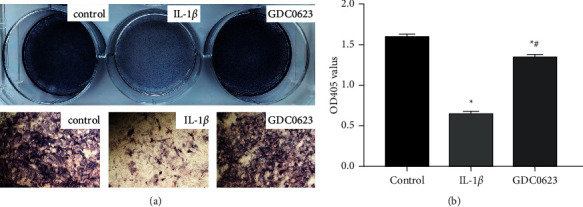
Comparisons of ALP staining and activities among the control, IL-1*β*, and GDC0623 groups. (a) Osteoblasts were stained for ALP levels after being induced with an osteogenic differentiation medium for 7 days (upper lane). Details of ALP staining at day 7 (lower lane). (b) ALP activities were detected after cell induction for 7 days. Data are expressed as mean ± SD for *n* = 3. ^*∗*^*p* < 0.05 versus the control group; ^#^*p* < 0.05 versus the IL-1*β* group.

Compared to the control group, after 7 days of osteogenic induction, ALP staining showed that signals in IL-1*β*-treated groups were significantly inhibited. However, the ALP signal recovered after GDC0623 treatment (Figure 2(a)). ALP activities were further quantitatively analyzed, showing that ALP activities of osteoblasts in the IL-1*β* groups were lower than those of the control group. However, ALP activities in osteoblasts significantly recovered after GDC0623 treatment (Figure 2(b)).

### 3.3. Effects of GDC0623 on Mineralized Nodule Formation and Calcium Influx in Induced Osteoblasts

**Figure 3:**
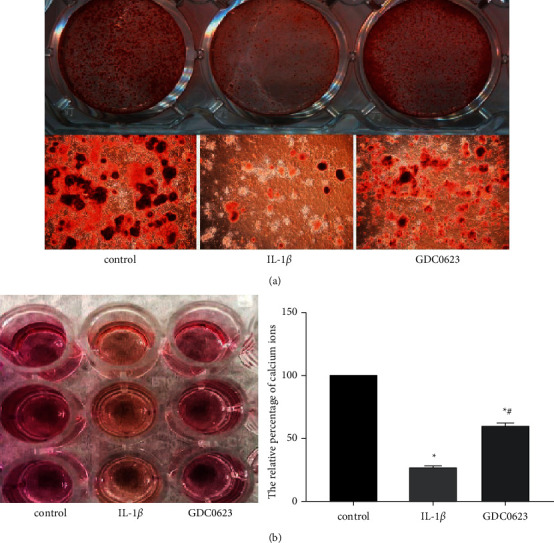
GDC0623 treatment affected calcified nodule formation and intracellular calcium levels. (a) Mineralized nodules were stained after osteoblast induction in the osteogenic differentiation medium for 21 days (upper lane), the details of mineralized nodules (lower lane). (b) Intracellular calcium levels were assessed by the calcium assay kit after treatment with the differentiation medium for 7 days. Data are expressed as mean ± SD for *n* = 3. ^*∗*^*p* < 0.05 versus the control group, ^#^*p* < 0.05 versus the IL-1*β* group.

Osteoblast differentiation was a prerequisite to bone formation. Mineralized nodules were a late-stage indicator of bone formation activity. Observation of mineralized nodules of osteoblasts was one of the most commonly utilized means to study osteoblast differentiation. To investigate the impact of GDC0623 on the mineralization potential of osteoblasts, calcified nodule formation was assessed by Alizarin Red-S staining after 21 days of osteogenic induction. We established that calcified nodules exhibited low counts and occurred in smaller aggregates in IL-1*β* treated groups, compared with the control group. However, calcified nodules were recovered in the GDC0623 treatment group (Figure 3(a)).

Moreover, intracellular Ca^2+^ levels are potential markers for osteoblast differentiation and proliferation [17]. Cells were incubated with the induction medium for 21 days and lysed in a lysis buffer. Calcium levels were analyzed by quantifying the protein and adjusting to the same amount of protein in each sample. Results showed that intracellular calcium levels were suppressed in the IL-1*β* group, but increased after GDC0623 treatment (Figure 3(b)). The results described above suggested that inflammatory milieu could reduce calcium influx and inhibit osteoblast differentiation, which could be significantly reversed by GDC0623.

### 3.4. IL-1*β* Treatment Significantly Suppressed Osteoblast Gene Expressions

**Figure 4:**
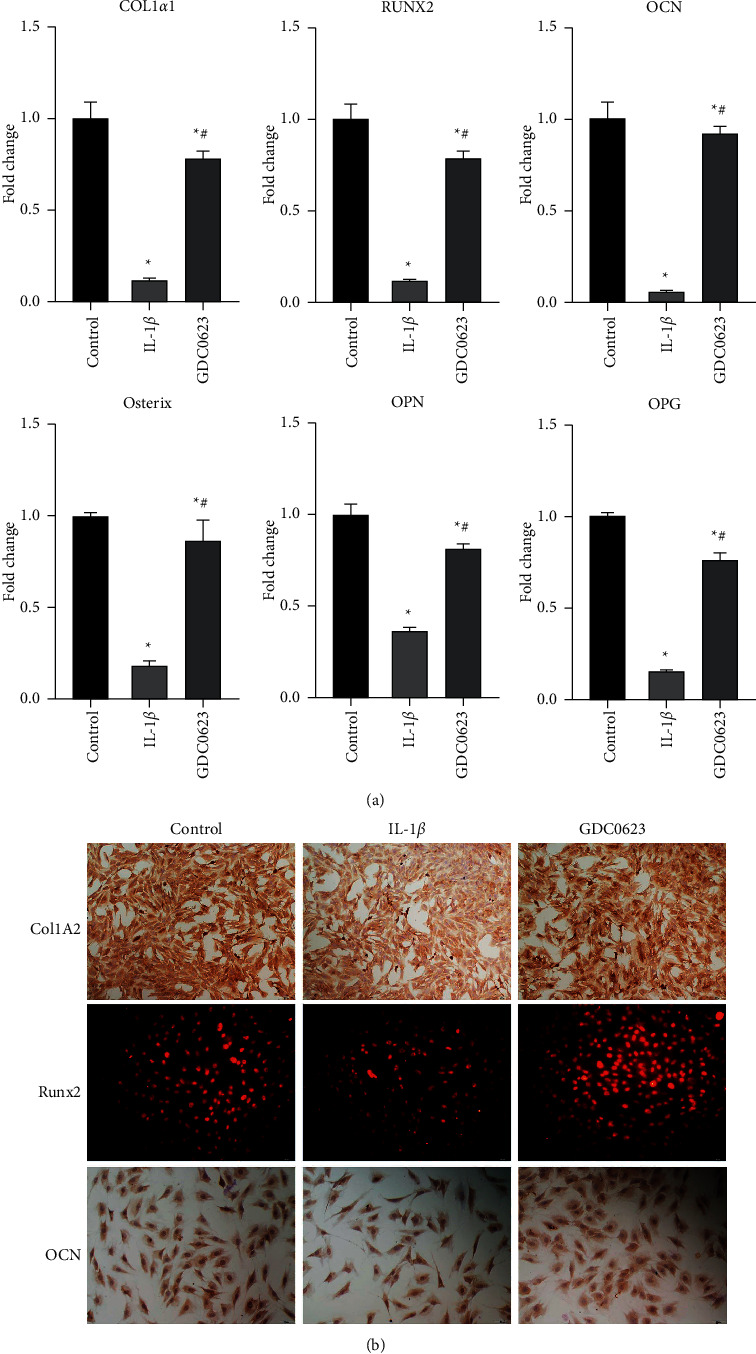
Osteoblastic gene expression profiles in osteoblasts after treatment with GDC0623. (a) Osteoblasts were maintained in an osteogenic differentiation medium containing IL-1*β* or IL-1*β* plus GDC0623 for 7 days. qPCR analysis of osteogenic gene expression in osteoblasts was performed, relative to internal control GAPDH and normalized to control cells. (b) Immunocytochemical staining of Runx2, OCN, and Col I in osteoblasts after treatment with IL-1*β* or GDC0623 plus IL-1*β* for 7 days. Data are expressed as mean ± SD for *n* = 3. ^*∗*^*p* < 0.05 versus the control group, ^#^*p* < 0.05 versus the IL-1*β* groups.

Expression levels of osteoblastic genes were evaluated by quantitative PCR to verify the effects of IL-1*β* on osteoblastic differentiation. Results revealed that IL-1*β* treatment significantly suppressed the expression levels of osteogenic markers, Col1a1, OCN, Runx2, Osterix, OPN, and OPG. These findings imply a significant inhibitory role of IL-1*β* in osteoblastic differentiation. The downregulated expressions of these genes were reversed by GDC0623 treatment (Figure 4(a)).

Analyses of protein expression levels of Col I (the principal component of bone matrix), Runx2 (a master transcription factor in osteogenic differentiation), and osteocalcin (primarily expressed in later stages upon mineralization) validated the mRNA expressions of osteoblastic genes (Figure 4(b)).

### 3.5. GDC0623 Blocked IL-1*β* Mediated Osteoblast Proliferation

**Figure 5:**
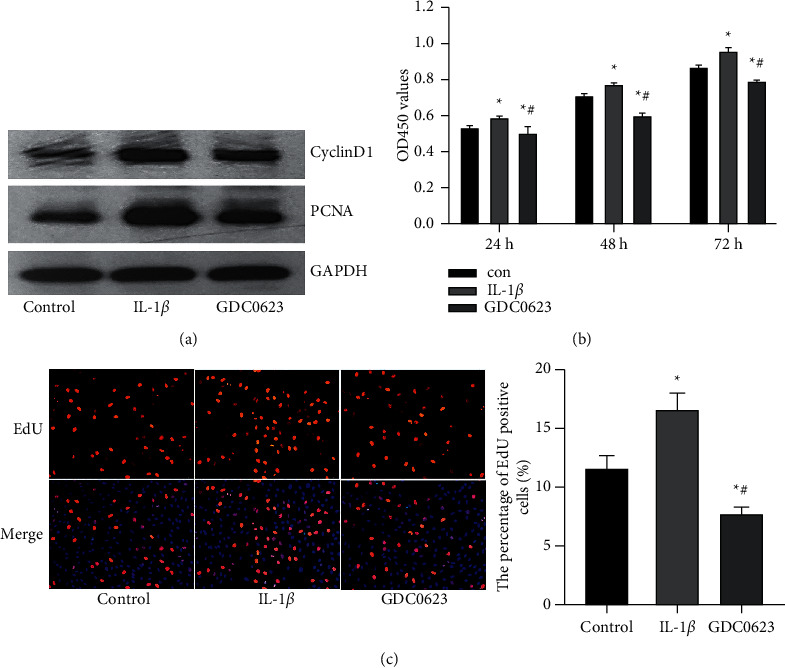
Effects of GDC0623 on osteoblast proliferation were evaluated by protein expression, CCK-8, and EdU staining assays. (a) Expression levels of PCNA and CyclinD1 were investigated by western blot. (b) Osteoblasts were treated with IL-1*β* or IL-1*β* plus GDC0623 for 24 h, 48 h, and 72 h. Optical densities of the three groups at 450 nm were determined and analyzed. (c) EdU incorporation assay of the three groups is on the left and statistical analyses of EdU positive cells are on the right. Data are expressed as mean ± SD for *n* = 3. ^*∗*^*p* < 0.05 versus the control group, ^#^*p* < 0.05 versus the IL-1*β* group.

Upregulated expression levels of PCNA and Cyclin D1 protein indicated the treatment of osteoblasts with IL-1*β* promoted cell growth (Figure 5(a)). The CCK8 assay further revealed the proliferative effect of IL-1*β* on osteoblasts (Figure 5(b)). However, IL-1*β*-induced osteoblast proliferation significantly decreased after GDC0623 treatment. In the EdU assay, EdU-positive cells (stained in red) were the proliferated cells, while DAPI stained cells (stained in blue) were the total cells (Figure 5(c), left). The proportion of EdU positive cells was consistent with results of the CCK8 and western blot assays (Figure 5(c), right). These results suggest that GDC0623 inhibited IL-1*β*-induced osteoblast proliferation.

### 3.6. GDC0623 and C188-9 on MEK-ERK1/2 and Jak-Stat3 Signaling Pathways as Well as on Expression Levels of MMP9 and MMP13 in IL-1*β*-Treated Osteoblasts

**Figure 6:**
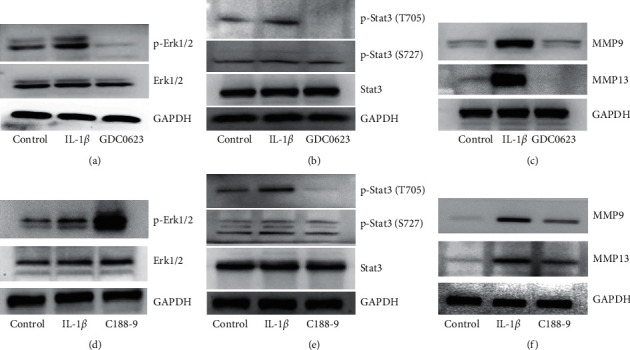
Effects of GDC0623 and C188-9 on MEK-Erk1/2 and Jak-Stat3 signaling pathways and on expression levels of MMP9 and MMP13. Osteoblasts were pretreated with DMEM plus 0.1% FBS overnight and intervened with IL-1*β*, IL-1*β* plus GDC0623, and IL-1*β* plus C188-9 for 60 min. (a–c) Protein levels of p-Stat3, P-Erk1/2, MMP9, and MMP13 were analyzed after IL-1*β*-stimulated osteoblasts had been treated with GDC0623. (d–f) Expression levels of p-Stat3, P-Erk1/2, MMP9, and MMP13 were assessed by blocking the Jak-Stat3 pathway with C188-9.

Levels of phosphorylated proteins in MEK-Erk1/2 and Jak-Stat3 signaling pathways were evaluated by western blot assays after IL-1*β*, IL-1*β* plus GDC0623, or IL-1*β* plus C188-9 treatment for 60 min. Expression levels of p-Stat3 (T705) and p-Erk1/2 were upregulated in the IL-1*β* group, but downregulated in the GDC0623 group (Figures 6(a) and 6(b)). This indicated that activities of MEK-Erk1/2 and Jak-Stat3 were inhibited. The Stat3 inhibitor, C188-9, promoted Erk1/2 activation and inhibited p-Stat3 (T705) (Figures 6(d) and 6(e)). Expression levels of MMP9 and MMP13 were elevated in the IL-1*β* group, but were suppressed upon GDC0623 and C188-9 treatment (Figures 6(c) and 6(f)).

### 3.7. The Stat3 Inhibitor, C188-9, Suppressed Osteogenic Differentiation Independent of MMP9 and MMP13 after IL-1*β* Stimulation

**Figure 7:**
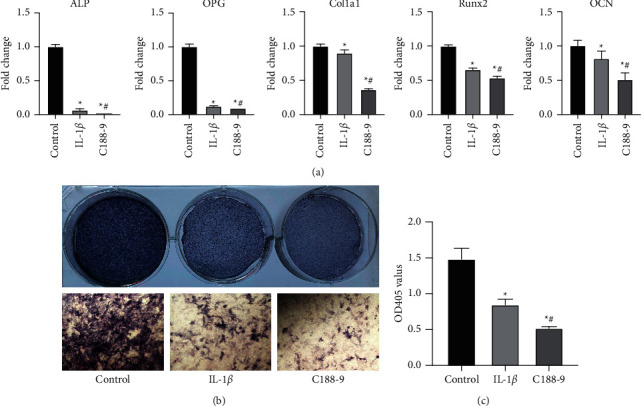
Effects of C188-9 on expressions of osteogenic marker genes, ALP staining, and ALP activities. Osteoblasts were cultured in an osteogenic induction medium containing IL-1*β* or IL-1*β* plus C188-9 for one week. (a) qPCR analysis of osteogenic gene expressions in osteoblasts was performed, relative to internal control GAPDH, and normalized to control cells. (b) ALP staining (upper) and details of staining (lower) among the three groups. (c) Data are expressed as mean ± SD for *n* = 3. ^*∗*^*p* < 0.05 versus the control group, ^#^*p* < 0.05 versus the IL-1*β* group.

Elevated expressions of MMP9 and MMP13 after IL-1*β* treatment were downregulated by C188-9 treatment. Osteoblasts were treated with IL-1*β* or IL-1*β* plus C188-9 to confirm the effects of c188-9-induced MEK-Erk1/2 activation on osteogenic differentiation. Osteogenic differentiation was further determined by assessment of ALP staining (Figure 7(b)), ALP activities (Figure 7(c)), and expressions of osteogenic marker genes (Figure 7(a)). These results show that MEK-Erk1/2 activation plays a critical role in osteogenic differentiation.

## 4. Discussion

The MEK-ERK1/2 signal pathway contributes to the development of many human cancers. The MEK inhibitor, trametinib, has been shown to reduce neuroinflammation and suppress tumor progression [18]. Trametinib also exerts anti-inflammatory effects in rheumatoid arthritis (RA) [19]. Some researchers have shown that AZD6244 inhibits acrolein-induced neuroinflammation [20]. This indicates that these anticancer drugs can potentially benefit patients with inflammatory-related diseases. GDC0623, a highly selective non-ATP competitive MEK inhibitor, was found to suppress tumor development [21].

Currently, it is not well understood whether GDC0623 can inhibit inflammation in diseases such as OP. Clinically, OP presents as a complex disease associated with chronic inflammation. In the present study, the effects of GDC0623 on the osteoblastic differentiation in the context of inflammation were investigated.

The overall effect of different concentrations of IL-1*β* on osteogenesis was explored. The results showed that treatment with IL-1*β* inhibited ALP activity in a dose-dependent manner. Notably, the lowest activity of ALP in osteoblasts was recorded at IL-1*β* concentration of 20 ng/ml. Therefore, this dose was subsequently used to establish an inflammatory model of osteoblasts undergoing osteogenic differentiation.

The effect of IL-1*β* or IL-1*β* combined with GDC0623 on osteogenesis was examined. Generally, osteoblast differentiation can be divided into three stages: (1) osteoblasts proliferation, (2) osteoblasts differentiation, and (3) osteoblasts maturation. Alkaline phosphatase (ALP) is an early marker of osteogenic differentiation when the cells are undergoing mineralization [22]. Extracellular matrix (ECM) mineralization is the final stage of osteogenic differentiation. Results show that IL-1*β* treatment suppressed the staining intensity of ALP and activity. Similarly, treatment with IL-1*β* for 21 days decreased the size and number of mineralized nodules as revealed by Alizarin Red staining. However, the effect of IL-1*β* on ALP activity and formation of calcified nodules was reversed by GDC0623. The formation of mineralized matrix is influenced by extracellular levels of calcium. In addition, extracellular levels of calcium are important determinants of intracellular calcium homeostasis which is regulated by calcium sensing receptor (CaSR) and voltage-dependent calcium ion channels [23]. Exposure of osteoblasts to osteogenic induction medium containing IL-1*β* decreased the concentration of calcium, and this effect was abolished by GDC0623 treatment.

The results of ALP activities and mineralized nodules were validated via quantitative analysis of all these osteogenesis-related genes in osteoblasts after osteogenic induction for one week. Type I collagen secretion plays a crucial role in osteogenic differentiation. Moreover, Col1a1 is a significant factor in bone formation and is a potential marker for osteogenesis differentiation [24]. During osteoblastic differentiation, Runx2 stimulates the transcription of important differentiation-associated downstream target genes, including those encoding osteocalcin (OCN) and OPN. Elevated OCN and OPN levels are associated with late stages of differentiation and mineralization [25]. In this study, GDC0623 treatment successfully reversed IL-1*β*-mediated inhibitory effects on expressions of osteogenic markers. This finding implies that GDC0623 treatment reverses IL-1*β*-induced inhibition of osteoblast.

In this experiment, IL-1*β* markedly stimulated rat osteoblast proliferation, which was inhibited by GDC0623 treatment. Elevated osteoblast proliferation levels enhance bone formation during normal bone remodeling [26]. However, IL-1*β* treatment inhibited osteogenic differentiation. These findings suggest that osteoblast proliferation did not play a major role in regulating osteogenic differentiation.

In a previous study, the atp6v1h zebrafish mutant showed a serious reduction of mature calcified bone cells quantitatively and a significant increase in expressions of MMP9 and MMP13. However, suppression of MMP9 and MMP13 levels rescued bone density defects [27]. Moreover, during bone development, MMP9-null and MMP13-null mice exhibited expanded hypertrophic zones and osteopetrosis [28, 29]. Compared to single knockouts, double knockout of MMP9 and MMP13 significantly expanded the length of the hypertrophic zone. This suggests a synergistic relationship between MMP9 and MMP13 [30]. In this study, IL-1*β* treatment upregulated MMP13 and MMP9 levels in osteoblasts, both of which were downregulated by GDC0623 treatment. MMP13 and MMP9 protein levels are associated with osteogenic differentiation capacities.

The signal transducer and activator of transcription 3 (Stat3) signaling pathway plays a vital role in bone homeostasis. In mice, suppressed Stat3 pathways in osteoblasts and osteoclasts significantly reduced bone mineral densities [31, 32]. Moreover, inactivated Stat3 in osteoblasts and osteocytes has been associated with mechanical load-driven bone formation [33]. Cellular hypoxia promoted osteogenic differentiation by upregulating Stat phosphorylation in MSCs [34, 35]. An increase in Stat3 phosphorylation has a beneficial role on bone defect healing [36]. In mesenchymal stem cells, Stat3 activation was shown to enhance osteogenic differentiation as well as *in vivo* bone formation [37]. Moreover, upstream of STAT3 signaling, upregulated JAK2 induces osteogenic differentiation of progenitor cells and bone defect healing [38].

In this study, phosphorylation levels of Stat3 and Erk1/2 were elevated in IL-1*β*-treated osteoblasts; however, these effects were reversed by GDC0623 treatment. In addition, IL-1*β*-activated Stat3 did not improve osteogenic differentiation. These findings contrast with those of a previous study. We found that GDC0623 treatment suppressed Stat3 phosphorylation but promoted osteogenic differentiation. The effects of the Stat3 specific inhibitor (C188-9) on osteoblastic differentiation and activities of the MEK-Erk1/2 pathway were investigated to verify the functions of Stat3. Relative to the IL-1*β* group, expression levels of MMP9 and MMP13 were also inhibited in C188-9-treated osteoblasts. C188-9 treatment stimulated MEK-Erk1/2 activation but suppressed osteogenic differentiation.

Expression levels of MMP9 and MMP13 were respectively downregulated in GDC0623- and C188-9-treated osteoblasts while osteogenic differentiations were totally different. These findings imply that during osteogenic differentiation under inflammatory conditions, MMP9 and MMP13 are not downstream molecular targets of MEK-Erk1/2 and Jak-Stat3 pathways. Our conclusions differ from those of previous studies. Differences in outcomes could have been due to various factors. In previous studies, the roles of MMP9 and MMP13 were evaluated with regard to bone development.

We also found that IL-1*β*- or C188-9-induced MEK-Erk1/2 activation suppressed osteogenic differentiation, implying that MEK-Erk1/2 activation plays a crucial inflammatory role during osteogenic differentiation. Therefore, we postulated that IL-1*β* activated the MEK-Erk1/2 inflammatory response pathway. MEK-Erk1/2 activation significantly inhibited osteogenesis, which was reversed by GDC0623 treatment. These findings imply that GDC0623 is an anti-inflammatory compound with the potential for treating OP.

## 5. Conclusion

The proinflammatory cytokine, interleukin-1*β*, plays a key role in inflammatory responses by suppressing bone formation of osteoblasts or increasing bone absorption of osteoclasts, which led to osteoporosis. In this study, IL-1*β* exerted these effects by activating the MEK-Erk1/2 inflammatory response pathway; however, GDC0623 treatment significantly reversed the inhibitory effects of IL-1*β* on osteogenic differentiation.

## Figures and Tables

**Table 1:** Primers used for real-time PCR.

Gene name	GenBank accession no.	mRNA sequences (5′-3′)
GAPDH-F		TTCAACGGCACAGTCAAGG
	NM_017008.4	
GAPDH-R		CTCAGCACCAGCATCACC
OCN-F		CCCAATTGTGACGAGCTAGC
	M25490.1	
OCN-R		CTGTGCCGTCCATACTTTCG
Runx2-F		AAGGAGCACAAACATGGCTG
	NM_001278483.1	
Runx2-R		TCTTAGGGTCTCGGAGGGAA
Osterix-F		CAAHGGTTAGGTGGTGGGC
	AY177399.1	
Osterix-R		TCTTGGGGTAGGACATGCTG
OPN-F		CGGTGAAAGTGGCTGAGTTT
	M14656.1	
OPN-R		GGCTACAGCATCTGAGTGTTTG
OPG-F		TGAGGTTTCCAGAGGACCAC
	U94330.1	
OPG-R		GGAAAGGTTTCCTGGGTTGT
COL1*α*1-F		CATGTTCAGCTTTGTGGACCT
	NM_053304.1	
COL1*α*1-R		GCAGCTGACTTCAGGGATGT
ALP-F		GCACAACATCAAGGACATCG
	NM_013059.1	
ALP-R		TCAGTTCTGTTCTTGGGGTACAT

## Data Availability

All data generated or analyzed during this study are included in this published article.
